# Change in Refractive Error Associated With the Use of Cannabidiol Oil

**DOI:** 10.7759/cureus.14434

**Published:** 2021-04-12

**Authors:** Amir Ali, Praveena K Gupta

**Affiliations:** 1 Department of Ophthalmology and Visual Sciences, University of Texas Medical Branch, Galveston, USA

**Keywords:** myopia, hyperopic shift, cannabidiol oil, diabetes mellitus

## Abstract

Cannabinoid (CBD) products have gained popularity since their legalization in 2018, causing a plethora of unregulated CBD products to be sold in the United States. These products are available in various combinations for topical and oral consumption, claiming credit for potentially improving various diseases. In this report, we present a newfound case reporting a shift in refraction that may be associated with the regular use of CBD oil supplements.

A 57-year-old woman with a history of diabetes mellitus type 2, hyperlipidemia, obstructive sleep apnea, with no change in medications, diet, or lifestyle was found to have a hyperopic shift in vision with the recent daily addition of CBD oil intake.

This case report highlights the possible association of CBD oil and vision changes after regular consumption of CBD oil in an otherwise stable patient. Further study is required to understand the mechanisms of CBD oil-associated shift in refractive error. Because the patient is diabetic and the refraction shift was hyperopic, other etiologies, such as un-noted lenticular change, cannot be ruled out. CBD products are unregulated and marketed in many mixed forms, and thus can cause unforeseen effects on susceptible individuals. This warrants Food and Drug Administration (FDA) regulation of such products and extensive research before considering them for therapeutic usage.

## Introduction

Cannabis, or marijuana, has been utilized in psychogenic therapy for hundreds of years. Over the past few decades, it particularly has a newfound use in pain medicine, neurology, oncology, gastroenterology, and ophthalmology [1-2]. Recently, cannabinoid (CBD) oil has been immensely used as supplements and in beverages after the passing of the Hemp Farming Act (HFA) in 2018, which legalized hemp-derived products in the United States. This has allowed commercial companies to produce and sell unregulated CBD oil products without US Food and Drug Administration (FDA) approval. Each product of CBD is potentially unregulated as an uncontrolled substance with varying concentrations, variance in the quality of hemp varieties, and lipid oxidation profiles [3]. Without regulation and medical guidance, CBD oil products can cause severe side effects [4]. We present an isolated case of a patient who reported gradual improvement in myopic vision after starting cannabidiol (CBD) oil for the past few weeks and reversal to original myopic refraction after the discontinuation of CBD oil. To our knowledge, this is the only case report that presents a hyperopic shift in association with cannabidiol oil intake in the English language ophthalmic literature.

## Case presentation

A 57-year-old, white female presented to the optometry clinic with eye strain and a gradual decrease in her vision for the last three weeks. She reported her eye strain was somewhat relieved after she removed her glasses. Her medical history was remarkable for obstructive sleep apnea, hyperlipidemia, and polyneuropathy secondary to continued uncontrolled type 2 diabetes (most recent hemoglobin A1c = 12.8%), osteopenia, and restless legs syndrome. Her social history included cigarette smoking (seven cigarettes a day with a five-pack-year history). She denied the use of alcohol or recreational drugs. Her ocular history pertaining to trauma or any surgery was negative. Additionally, she noted having no other symptoms such as headache, dry eyes, double vision, vision loss, spots, or threads in her vision.

**Figure 1:**
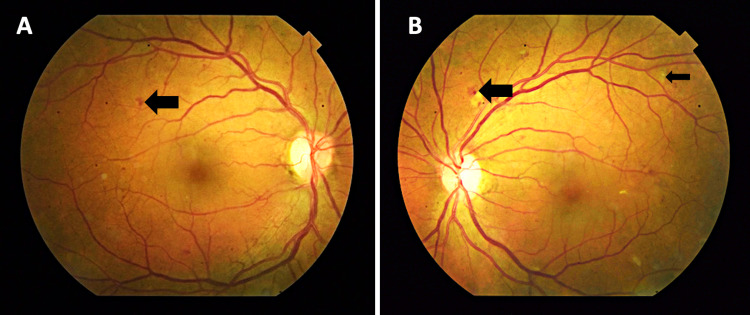
Color fundus photographs of the right eye (A) and left eye (B) with non-proliferative diabetic retinopathy Cotton wool spot (thin arrow), dot blot, and exudates (thick arrow)

**Figure 2:**
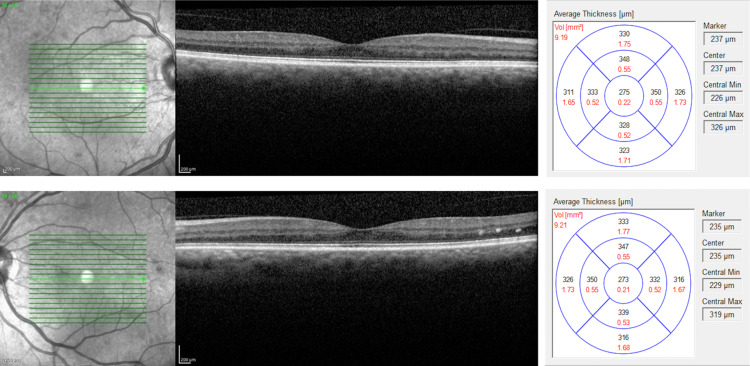
Optical coherence tomography images of the retina right eye (top) and left eye (bottom) Fovea shows normal contour with no central edema; average central macula thickness of 275 µM OD and 273 µM OS

On examination, her visual acuity (VA) with her habitual glasses was 20/60 in the right eye (OD) and 20/70 in the left eye (OS), pin holed to 20/40 OD and 20/40 OS. The pupils were round and reactive to light OU, with no relative afferent pupillary defect. External examination, extraocular muscle movements, and counting finger visual field tests were normal. Her intraocular pressure was 16 mmHg in the right eye and 17 mmHg in the left eye, measured with a tonopen. The dilated fundus examination revealed rare cotton wool spots, microaneurysm, dot-blot hemorrhages, and vascular attenuation consistent with moderate, non-proliferative diabetic retinopathy in both eyes without any signs of macular edema (Figure 1). Her optic cups appeared normal with no signs of glaucoma. Given her decrease in vision and clinical presentation of non-proliferative diabetic maculopathy, we decided to run a macular optical coherence tomography (OCT) scan (Figure 2). The fovea showed a normal contour, no central macular edema, and an average central retinal thickness of 275 µM OD and 273 µM OS.

On refraction, her manifest refraction had shifted from her habitual of -2.25 D sphere to -0.75 D in the right eye and from a habitual of -2.00 D to -0.75 D sphere in the left eye. Her new corrected VA in the right eye was 20/25 and 20/25 in the left eye, and a new pair of prescription glasses were made. This new information of hyperopic shift led us to systemically review her medications for possible associations.

Her medications included multivitamins, dulaglutide, canagliflozin, sitagliptin-metformin, lisinopril, gabapentin, pramipexole, clotrimazole-betamethasone, cyclobenzaprine, glucosamine sulfate, zolpidem, cetirizine, ranitidine, and magnesium oxide. In addition to the above, over the past eight weeks, she had started taking 750 mg of peppermint-flavored Full Spectrum CBD Oil (HempWorx, MyDailyChoice, Las Vegas, Nevada), 12 drops twice a day for restless leg syndrome. The patient reported having improved sleep but associated gradual blurry vision which made her visit the optometry clinic.

At the three and six-month follow-ups, the patient’s refraction was re-assessed, and her improved VA remained stable with no report of blurry or worsening of vision, headache, or eye strain. The patient continued to take CBD oil regularly as before and claimed her improved vision to the intake of CBD oil. The patient additionally reported no significant change in diet, lifestyle, and medication and reported her new glasses to be “perfect.” At the nine-month tele-visit follow-up, the patient ran out of CBD oil and thus had to stop taking CBD oil for three to four weeks. Within three weeks of stopping the CBD oil, the patient again noted a gradual worsening of her vision. The patient tried her old prescription lenses with -2.25 OD and -2.00 OS refractive error correction and reported seeing clearly. She had reverted to her original myopic state after stopping the CBD oil.

## Discussion

Based on the patient’s history and ocular examination, there is a clear hyperopic shift in the patient’s refraction after initiation of the CBD oil supplement and its reversal after stopping the CBD oil. Her refractive error remained unchanged while she was on the CBD oil supplement and attests to the use of CBD oil regularly as she finds relief from her restless leg syndrome. Possible etiologies of her refractive shift include the patient’s status of diabetes mellitus type 2, medication history, and her recent use of CBD oil [5].

Based on the exam and imaging, our patient had classic diabetic retinopathy (last HbA1C 12.8%) with no significant macular edema. Myopic shifts in vision are reported in about 4% of the diabetic patient population, however, hyperopic shifts are reported even less often. Myopic and hyperopic shifts have been traditionally thought to be due to hyperglycemia and hypoglycemia, respectively, but recent studies have suggested hyperopic shifts can also occur due to hyperglycemia [6]. The hyperopic type of refractive shift in an uncontrolled diabetic patient has been mostly attributed to the changes in the refractive index of the lens due to fluctuations in water distribution [7-9]. In the current patient, the timing of the onset of refractive change, along with her chronic state of uncontrolled diabetes (based on her last three years of high Hb A1C numbers), is unlikely due to her hyperglycemia, especially when associated with the change in vision over three weeks of time after the initiation of CBD oil. Interestingly, her refractive shift was stable while she was on the CBD oil and reverted to her original myopia after she stopped taking the CBD oil.

The association of vision change after the start of the CBD oil cannot be ruled out as one of the plausible causes. The authors are aware that this is an isolated case report where the patient clearly related her gradual change in vision after starting the CBD oil drops twice daily for six weeks. While the mechanism by which CBD oil affects refractive error is still an area for further exploration, CBD has been shown to regulate blood flow in retinal vessels and help in reducing neurotoxicity, oxidative stress, and blood-retinal barrier breakdown. The possible inhibition of p38 MAP kinase may also be a possible theory for the hyperopic shift [10-12]. Effects of cannabinoids on the anterior segment of the eye are also multiplex, and some studies indicate decreased corneal endothelial density [13]. Further studies will be needed to assert the findings from this isolated presentation of the case to better understand the role of CBD oil in refractive errors of the eye, especially in a diabetic condition.

## Conclusions

We present a case where a woman taking CBD oil orally for six weeks on a regular basis was found to have an improvement in myopia. In addition, her hyperopic shift reverted to her original myopic vision once she stopped taking her CBD oil. To our knowledge, this is the first case of CBD oil in association with a hyperopic shift. The mechanisms by which her VA improved are uncertain and can vary from possible corneal changes to retinal vasculopathy. Hyperopic shift due to her diabetes and antihistamine medications are a possibility, although unlikely, due to her established disease and chronic medication use. There is no previous case report of such an association in hyperopic shift and no prior head-to-head study looking at specific types of CBD oils and other forms of cannabinoid products. This novel isolated incident between CBD oil and change in VA requires additional, controlled, blinded research for further applicability.
